# COVID Chronicles: A Comics Anthology

**DOI:** 10.5195/jmla.2022.1369

**Published:** 2022-01-01

**Authors:** Tenley Sablatzky

**Affiliations:** 1 tsablatzky@gmail.com, Medical Librarian, Undergraduate Medical Academy, Prairie View A&M University, Prairie View, TX

*COVID Chronicles: A Comics Anthology* is a simultaneously heartwarming and terrifying collection of eighty-two unique voices sharing their perspectives on the trials and tribulations of living through the COVID-19 pandemic. The comics include perspectives through the eyes of frustrated children, exhausted medical professionals, im-munocompromised individuals, and many others.

Some of the comics are written as diary entries, such as Jason Chatfield's “COVID-19 Diary,” which tells the story of what happened when he and his wife were diagnosed with the coro-navirus. Others, such as Laura Holzman's “Isolation Exercises,” Lee Marrs's “Shelter-in-Place Sing,” and Rob Kraneveldt and Mike Garcia's “My New Normal: Rinse & Repeat,” showcase what self-isolation can do to one's mental health. “Quarantine Week 10” by Comic Nurse (MK Czerwiec) is a humorous take on a teacher trying their best to make do with virtual learning during an unprecedented time.

Kendra Boileau writes in the foreword: “Strange, perhaps, for these emotions to resonate so clearly in a medium that people often assume is either directed toward children or there for our amusement. But comics have a history of tackling weighty and mature subjects— and doing it well.” (p. ix).

For the past decade, scholars and medical education professionals have been debating the best practices for integrating the arts and humanities into medical education. Many scholars, including Danielle G. Rabinowitz in her 2021 article “On the arts and humanities in medical education,” stress the importance of using medical humanities as a tool for training empathic and compassionate physicians with well-rounded critical thinking skills.

Rabinowitz writes: “We are in a moment in medicine in which the demands on physicians are ever-increasing, as new technologies and scientific discoveries are rapidly changing the medical landscape. Rates of burnout are skyrocketing, as are studies showing that empathy among medical students is becoming progressively stunted” [1].

Never has the need for empathetic, well-rounded, and intelligent physicians been more crucial to the betterment of society. *COVID Chronicles* will be a fantastic addition to any library's medical humanities collection given its relevance to the current social, cultural, and political climate. The artists' stunning illustrations and relatable prose will at once make the reader laugh and cry, feel enraged and hopeful.
